# The Use of Transarterial Approaches in Peripheral Arteriovenous Malformations (AVMs)

**DOI:** 10.3390/jcm7050109

**Published:** 2018-05-09

**Authors:** Aditya Khurana, Patrick T. Hangge, Hassan Albadawi, M-Grace Knuttinen, Sadeer J. Alzubaidi, Sailendra G. Naidu, J. Scott Kriegshauser, Rahmi Oklu, Brian W. Chong

**Affiliations:** 1 Mayo Clinic School of Medicine, Mayo Clinic, Scottsdale, AZ 85259, USA; Khurana.Aditya@mayo.edu; 2 Department of General Surgery, Mayo Clinic, Phoenix, AZ 85054, USA; Hangge.Patrick@mayo.edu; 3 Division of Vascular and Interventional Radiology, Minimally Invasive Therapeutics Laboratory, Mayo Clinic, Phoenix, AZ 85054, USA; albadawi.hassan@mayo.edu (H.A.); Knuttinen.Grace@mayo.edu (M-G.K.); Alzubaidi.Sadeer@mayo.edu (S.J.A.); naidu.sailen@mayo.edu (S.G.N.); skriegshauser@mayo.edu (J.S.K.); 4 Department of Radiology and Neurological Surgery, Mayo Clinic, Phoenix, AZ 85054, USA; chong.brian@mayo.edu

**Keywords:** arteriovenous malformations, nidus, transarterial

## Abstract

Arteriovenous malformations (AVMs) are a subset of congenital vascular malformations (CVMs). They comprise abnormal connections between arterial and venous circulation; treatment approaches are dependent on the angioarchitecture of the AVM, specifically the number and arrangement of the feeder arteries and outflow veins. Various imaging modalities can be used to diagnose and plan treatment. Here we will review the use of transarterial approaches to treat AVMs.

## 1. Introduction

Arteriovenous malformations (AVMs) are a subset of congenital vascular malformations (CVMs), along with venous and lymphatic anomalies, according to the International Society for the Study of Vascular Anomalies (ISSVA) classification of vascular anomalies [1]. They can be located in multiple regions of the body and can be symptomatic and often challenging to treat, requiring a multidisciplinary approach [2,3]. Pathologically, AVMs refer to an abnormal shunting of blood between arterial and venous flow that bypasses an intervening capillary network. The anomalous region in which the arterial and venous vessels connect creates a low perfusion pressure, which is known clinically as the nidus [2]. AVMs can become progressively symptomatic up to adulthood [4]. As they progress, they can cause more severe manifestations, such as swelling, ulceration, hard-to-control bleeding, deformity, and even congestive heart failure due to strain on right-sided cardiac output [5]. Historically, AVMs were poorly defined using earlier treatment protocols due to the heavy reliance on only clinical presentation. With the advent of new technologies and a multidisciplinary classification system, physicians can provide a more accurate diagnosis at an earlier timeline, increasing the chances of the successful treatment of this type of CVM. There is an increased focus and emphasis on understanding the angioarchitecture of each patient’s AVM because the hemodynamics and extent of the AVM influence the treatment approach. AVMs can be treated endovascularly using three approaches—transarterial, transvenous, or direct cutaneous—with the goal of completely embolizing the nidus. Here, we will focus on the arterial treatment of arteriovenous malformations. Symptoms of AVMs can be location- and case-dependent, but AVMs around the body share many similar clinical manifestations and diagnostic treatments.

The authors recognize that transvenous and direct access techniques are also options. However, we will limit the scope of this manuscript to arterial approaches only.

## 2. Classification of AVMs

CVMs in general are divided into low-flow and high-flow vascular malformations. Low-flow malformations include lymphatic and venous malformations [6]. AVMs are a subset of high-flow CVMs that are further organized based on the morphology of the nidus.

It is important to determine the morphology and angioarchitecture of the AVM to minimize the risks of reflux of embolizing agents into normal arteries and veins. The Cho–Do classification of AVMs was developed to organize AVMs by their vascular structure. There are four categories that AVMs are organized into based on the structure of feeding and draining vessels of the nidus: types I, II, IIIa, and IIIb [7]. Table 1 provides a concise description of each morphological subtype.

Type I AVMs have an arteriovenous nidus structure in which three or fewer arteries shunt into a single draining vein. Typically this AVM can be treated using either a transarterial or transvenous approach [6].Type II AVMs have an arteriolovenous nidus structure in which multiple arterioles shunt blood into a single draining vein. This vein is usually much larger than the feeding arterial vessels, making it so this AVM type is best treated from the venous side with reflux of the embolic agent into the arteries feeding into the nidus. Both types of type III AVMs involve an abnormal connection between the arterioles and venules. Type IIIa has an arteriovenulous nidus structure without dilatation present, whereas Type IIIb has the same nidus structure with dilatation present. Type IIIa can only be treated via a transarterial approach, while type IIIb AVMs can be treated using transarterial, transvenous, or direct percutaneous approaches [7].

Wayne Yakes proposed a potential classification system in 2015, called the Yakes Classification System of AVMs [8]. The aim was to specifically associate treatment decisions with more specific AVM morphology patterns than the Cho–Do classification system. The utility is its usefulness in guiding treatments based on the angioarchitecture of the lesion, emphasizing not only the nidus but also the quantity and size of the draining veins. A description of the nidus types and treatment approaches for the Yakes AVM Classification System is summarized in Table 2. While the Yakes classification is predicated on the use of ethanol, some practitioners might use other liquid embolics such as ethylene vinyl alcohol copolymer (Onyx) and *n*-butyl cyanoacrylate (nBCA). It is important to note that the treatment approach for Type IV in this classification system can lead to severe tissue necrosis.

In a type Ia nidus, the goal is to directly occlude the fistulous communication between an artery and vein. Often this will involve the use of coils. Liquid embolics are less often used in this situation due to rapid flow through the fistula and difficulty achieving target embolization and occlusion without embolization through the venous side to nontarget locations [8].

Type IIa presents with a typical AVM nidus, and either transarterial access or direct puncture access is followed by embolization with alcohol or liquid embolics. A similar approach is taken with a type IIb nidus, where there is an aneurysmal draining vein present. The vein is usually coiled in addition to the transarterial approach describe for type IIa [8].

When there are multiple outflow veins, as is the case with type IIIb, each vein is coiled in addition to targeting the arterial supply with liquid embolics. Lastly, with type IV infiltrative AVMs, transcatheter or direct puncture is followed by embolization with alcohol due to its ability to penetrate small components of the AVM. In this setting, liquid embolics such as Onyx or nBCA are usually not as effective [8].

## 3. Clinical Presentation

AVMs are different from hemangiomas because they do not regress over time, instead growing at a physiologically normal rate, like the rest of the body. AVMs also differ from neoplastic disorders of the vasculature because they possess normal endothelial turnover rates [9].

**Figure 1:**
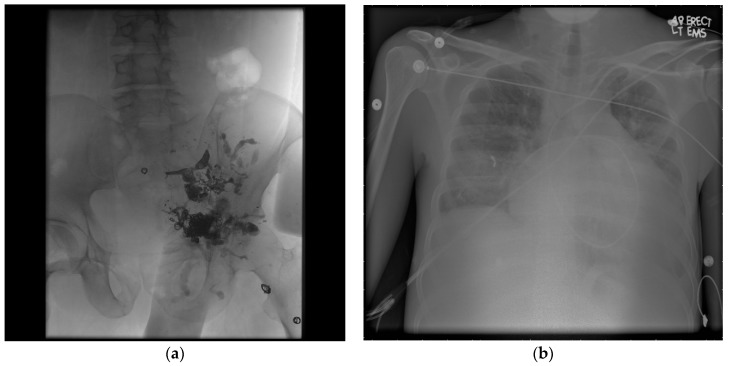
Schobinger Stage IV arteriovenous malformations (AVM). (**a**) The post-treatment angiogram shows the extent of a dilated hypogastric venous system due to drainage from the nidus and presence of a pelvic AVM with multiple outflowing veins as well as one particularly large flowing vein. The AVM has been treated from the venous side with coils and from the arterial side with a liquid embolic agent, indicative of a possible Yakes IIb/IIIb angiographic type. (**b**) A chest X-ray shows that the patient also presented with cardiomegaly and high-output cardiac failure due to stress on the venous circulation.

The Schobinger staging system, presented in Table 3, is currently used to classify AVMs based on their clinical presentation. Clinical signs of AVMs include dermatologic changes as well as cardiac irregularities in the most severe cases [10]. There are four stages within the Schobinger staging system. Stage I AVMs initially present with dermatologic symptoms, such as skin that possesses a cutaneous blush or warmth resembling a port wine stain [11]. Stage II AVMs present with more noticeable findings, such as bruits or thrills caused by circulation within the nidus as well as the presence of pulsatile masses. Stage III and IV involve pain and possible ischemic necrosis or ulceration of the tissue. Stage IV also involves high-output cardiac failure due to the irregularity in systemic circulation putting a strain on the right side of the heart [2]. Figure 1 shows an example of a stage IV AVM that resulted in high-output cardiac failure.

AVMs tend to worsen progressively with time, with 82.6% of stage I AVMs worsening before adulthood in a study by Liu et al., in which they observed the progression of 272 extracranial AVMs in children [12]. This underscores the importance of an accurate classification system and early diagnostic timeline.

Due to the abnormal connections between arterial and venous circulation, these vascular malformations can present with altered blood pressure compared to the normal contralateral part of the body [13]. AVMs can also clinically present with nerve injury, the ulceration of tissue, hemorrhage, and a reduction of limb function [3].

There has been some work into understanding the genetic etiology of AVMs. Perhaps a deficiency of transforming growth factor beta (TGF-β) leads to the development of these malformations [14,15]. Their expression has been linked previously to the expression of progesterone receptors during puberty [16]. If a patient presents with multiple AVMs concurrently, it is possible that familial syndromes are contributing to the patient’s clinical presentation. Two familial syndromes that are associated with AVMs are Parkes Weber and hereditary hemorrhagic telangiectasia [2]. Parkes Weber syndrome (PWS) is a congenital syndrome that can involve malformations of capillary, venous, lymphatic, and arteriovenous vessels. Arteriovenous malformations are the main benchmark that leads to diagnosis [17]. In a systematic review by Banzic et al., 31.3% of PWS cases analyzed presented with high-output heart failure due to AV shunting, indicating the presence of stage IV AVM [17]. Hereditary hemorrhagic telangiectasia is an autosomal dominant disorder with a prevalence of 1:5000 to 1:10,000 [18]. It can present with AVMs mainly in the pulmonary, cerebral, and hepatic circulation [18].

Not all AVMs prompt treatment, as the success of treatment is dependent on the highly individual angioarchitecture of each nidus. There are multiple indications to treat AVMs, both absolute and relative, shown in Table 4 [19]. If the AVM presents with severe symptoms such as hemorrhage, high-output cardiac failure, chronic venous hypertension, and lesions on limbs or in life-threatening locations, the treatment of AVM is necessary. Any disabling pain, functional disability, or cosmetic deformities can be relative indications of treatment, but further diagnoses from a multidisciplinary clinical team are recommended [19].

## 4. Diagnostic Imaging Modalities

It was previously difficult to manage AVMs based only on clinical manifestations. The development of imaging modalities has allowed providers to determine the angioarchitecture of the nidus to optimize treatment plans and minimize the risk of endovascular management. There are four types of minimally invasive imaging modalities that can currently be used to visualize the extent of an AVM before treatment: ultrasonography (US), magnetic resonance angiography (MRA), computed tomography angiography (CTA), and catheter angiography [2]. Typically AVMs will present as a high-density connection of vessels, indicating the presence of a nidus, with some presence of rapid shunting through the nidus [2].

US is a non-invasive imaging modality typically used as the initial workup to investigate any vascular malformation [20]. US can allow providers to determine whether the vascular malformation has characteristics of high-flow or low-flow CVMs [21]. AVMs in a US will typically show high systolic flow due to the pressure gradient of the nidus as well as shunting and spectral broadening. The nidus will present as a high-echogenicity area of vessels, perhaps due to the localized arterial and venous hypertrophy caused by the arterialized venous vessels receiving shunted blood directly from arterial connections [21]. However, the US technique does have some drawbacks. It is difficult to identify soft tissue and bony landmarks as well to further characterize vascular malformations according to their subtypes. US would not be useful in evaluating deep or complex AVMs [21]. Complex AVMs refer to vascular malformations that have multiple feeding and draining vessels connected to the nidus.

Color Doppler imaging is another important noninvasive technique for diagnosing and assessing vascular malformations. Specifically for high-flow AVMs, color Doppler can assess arterial flow rates before and after AVM treatment to determine the success of the procedure [3]. Typically, AVMs present with a turbulent multidirectional flow of blood, which would be evident on a color Doppler [2].

Typically, MRA with or without CTA is performed if the history and US findings are consistent with the diagnosis of an AVM [21]. MR has become the standard imaging tool to further distinguish and characterize the different subtypes of vascular malformations. MR allows clinicians to see the involvement of the AVM with surrounding soft tissue and bone, allowing them to foresee any complications from treatment such as tissue damage, ischemic necrosis, bleeding, and infection. Additionally, the temporal resolution of MRI angiography allows the identification of the nidus. AVMs will appear as a collection of vessels with rapid shunting of the contrast material through the nidus [21].

**Figure 2:**
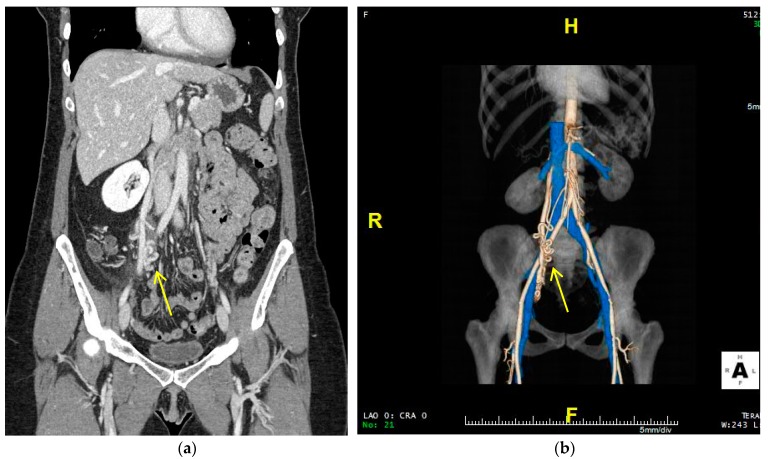
CT and computed tomography angiography (CTA) images of pelvic AVM. (**a**) The CT image on the left shows a conglomerate of tortuous vessels. (**b**) The CTA image on the right allows for better spatial resolution to visualize AVM morphology. Both modalities show a potential Cho–Do type II nidus with abnormal feeder connections from the infrarenal abdominal aorta and anterior division of the interior iliac artery and abnormal drainage to the right gonadal vein.

CTA has a higher temporal resolution than MRA, but is not as commonly used because of the ionizing radiation risk to the patient. Improvements in MRI and MRA have made it so that CTA is reserved for patients unable to undergo MRA or who need to be evaluated for acute symptoms, such as pulmonary complications [2]. The AVM in CT presents as a high-density area of vessels with the rapid shunting of the contrast material in the nidus [2]. These CT images can be reconstructed to create a 3D rendering of the regions with contrast enhancement [22]. This can allow for the specific spatial and anatomical evaluation of the AVM and perhaps help plan a more successful treatment approach. CTA can allow providers to better understand the anatomic variations for each patient and allow for more individualized and effective treatment [23]. Figure 2 shows an example of CTA and reconstructed 3D images of a pelvic AVM (Figure 2).

**Figure 3:**
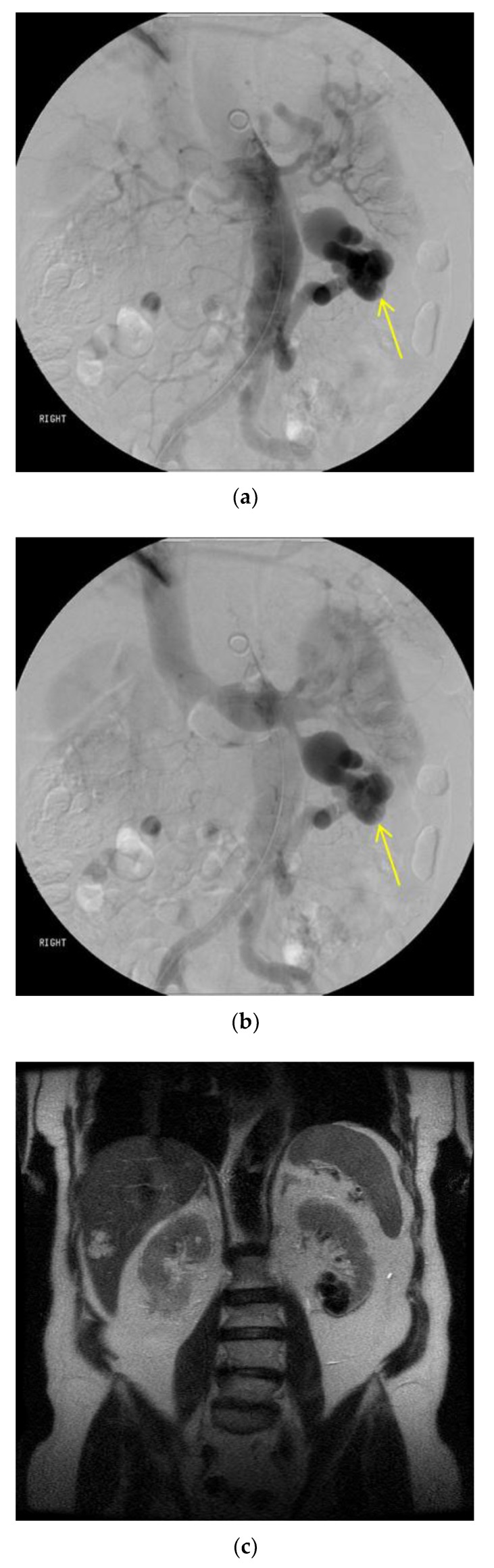
Catheter angiography of renal AVM. A nidus is detectable on catheter angiography by being visible on both arterial and venous phases of circulation. The AVM was located within the lower pole of the left kidney. The single feeder artery was from the distal abdominal aorta originating below the inferior mesenteric artery. There was a single draining vein into the left renal vein. This indicates a single shunt between arterial and venous circulation, Yakes type Ia or Cho-Do type I. (**a**) The image shows the presence of a nidus during the mid-arterial phase. (**b**) The image shows the nidus present in the mid-venous phase. (**c**) A magnetic resonance angiography (MRA) image confirms the involvement of the renal parenchyma in the area of the AVM, with a cystic lesion present in the lower pole of the left kidney, proximal to the site of the AVM.

Catheter-mediated angiography is the standard evaluation technique during procedures. It can determine more specifically the flow dynamics and angioarchitecture of the nidus, allowing the optimization of treatment approaches in real time. It is especially important for post-treatment follow-up because it can be used to confirm the presence of recurrence in the nidus [2]. Figure 3 shows the use of catheter angiography intraoperatively to determine the extent of a renal AVM.

## 5. Arterial Treatment of AVMs

The overall goal of the endovascular treatment of AVMs is to completely occlude or embolize the nidus such that abnormal shunting of blood through the nidus completely stops. Any of the three approaches to treat an AVM—transarterial, transvenous, or direct percutaneous—deliver a sclerosing or occlusive agent directly to the nidus that triggers immense endothelial inflammation and induces thrombosis formation at the site of the nidus, completely blocking the dense vessel network. Arterial treatments of AVMs can directly embolize the nidus or act as a supplementary or preliminary treatment to the nidus, so that direct cutaneous or transvenous approaches have a higher probability of success in occluding the low-pressure cell [24,25,26,27].

### 5.1. Embolotherapy of AVMs

Embolotherapy typically uses a transarterial approach to occlude the nidus [10]. Nevertheless, the treatment approach depends on the angioarchitecture of the AVM as well as the morphologic subtype of the nidus. This underscores the importance of accurate workup and diagnostic imaging. Certain morphological structures make it more difficult for a transarterial approach to have any therapeutic effect in reducing or occluding the AVM. Type II AVMs, in which there are multiple arterioles shunting blood into a single large draining vein, cannot be treated effectively with a transarterial approach. The therapeutic yield of the arterial treatment of Type II AVMs has been minimal [6]. Instead, direct puncture of the nidus or transvenous treatment with or without coil embolization achieves better results. This is due to the dominance of venous outflow when compared to the smaller feeding arterioles into the nidus [6]. Moreover, transarterial approaches to AVMs are typically not used when the nidus is proximal to normal branching arteries. Instead, a direct cutaneous or transvenous approach is used to prevent the migration of embolic agents into normal arterial circulation [10]. Additionally, transarterial approaches are difficult when feeding arteries are severely tortuous [28].

There are various tools for embolic treatment that can be used, depending on the size, complexity, and morphology of the AVM. Embolization can occur via the use of vascular plugs (Amplatzer; St. Jude Medical, St. Paul, MN, USA), *n*-butyl cyanoacrylate (nBCA) glue, coils, 98% ethanol, and the Onyx system [2].

### 5.2. Embolic Agents

Liquid embolic agents are relied on heavily in order to successfully treat AVMs. The two agents that are frequently used are Onyx (ethylene vinyl alcohol copolymer) and *n*-butyl cyanoacrylate (nBCA).

**Figure 4:**
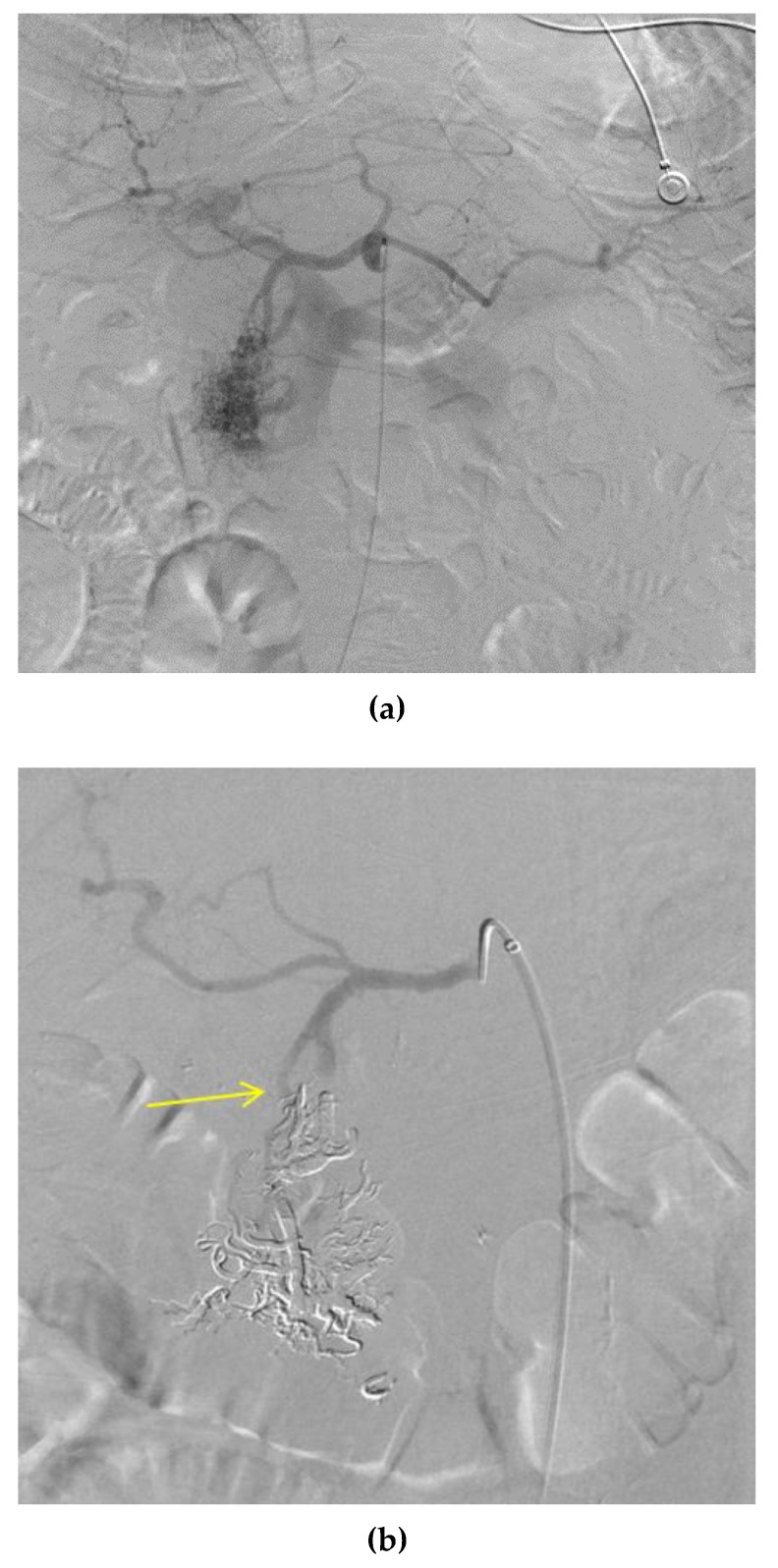

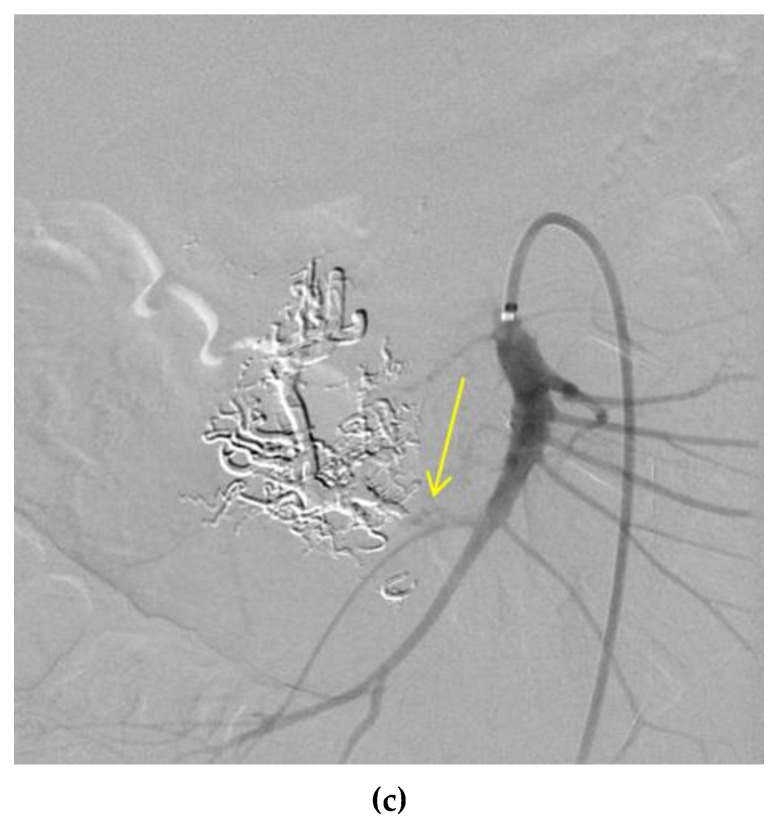
Embolization of a pancreatic AVM. Onyx is used to occlude a nidus with feeder arteries from both the gastroduodenal and superior mesenteric (SMA) arteries. Draining vessels include the superior mesenteric vein (SMV) and portal veins. (**a**) The image shows the presence of a spongiform nidus, with a “blush” of arteriole-venule connections, indicative of a Cho–Do type IIIa or Yakes type II AVM. (**b**) The image from a common hepatic artery digital subtraction arteriogram (DSA) shows the occlusion of gastroduodenal artery feeders; and (**c**) The image from an SMA DSA shows occlusion of SMA feeders. The liquid embolic agent has hardened and is dense (subtracted in these images) due to the addition of tantalum powder for radiographic visualization.

The development of the Onyx system has been important in the treatment and management of AVMs, particularly brain AVMs. Onyx is an embolizing agent (Micro Therapeutics, Inc., Irvine, CA, USA) created by dissolving ethylene—vinyl alcohol in dimethyl sulfoxide [29]. It promotes endothelial inflammation, causing intense thrombosis that will occlude the vessel [30]. Onyx was first only approved for the treatment of brain arteriovenous malformations, but since then been utilized to treat AVMs in many regions of the body. However, it is still mainly utilized in the treatment of cerebral and dural AVMs. The current literature shows cases where Onyx was used to embolize AVMs in the brain [29,31], mandible [32], kidney [33], pelvis [34], and liver [35]. Figure 4 shows the use of Onyx to embolize a pancreatic AVM (Figure 3). The disadvantages of Onyx include high costs, lengthy preparation time before procedure, and the necessity for extensive training on part of the interventionalist before use. The catheter could also become adhered to the Onyx within the AVM if not handled with the required technical expertise [36]. Furthermore, there has been some recent research that found that Onyx is not a particularly curative agent. In preliminary work by Meek et al., 13 of 18 post-Onyx embolization specimens showed signs of recanalization [37].

**Figure 5:**
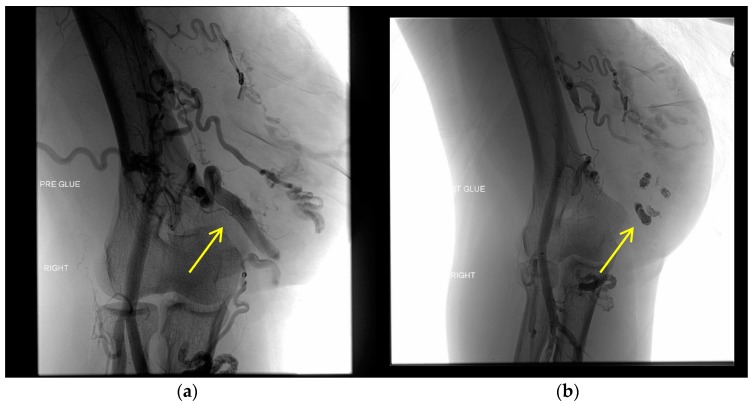
Embolization of an upper extremity AVM. Nidus formation (yellow arrows) with feeders from the brachial artery are occluded using the liquid embolic agent nBCA. The images show what appears to be a mixed AVM with proximal branches from the brachial artery and several large draining veins that eventually drain into the basilica vein, indicating a possible Yakes type IIa/IIb or Cho–Do type II/IIIa AVM. (**a**) Left image from a right brachial artery DSA shows the presence of the nidus pre-embolization. (**b**) Right image shows the occlusion of the nidus with nBCA, with added density using ethiodized oil, subtracted from this post-embolization DSA.

nBCA is an adhesive glue that begins to polymerize immediately when exposed to the anionic environment of the blood [38]. It works instantly to form a caste of the vessel and cause acute inflammation of the endothelial cells, prompting thrombosis and eventually fibrosis [39]. There is some risk of distal or reflux embolization that would immediately occlude non-target vessels [39]. Though used in many cases to manage and occlude AVMs, it can be difficult to control how much of the nidus is occluded, potentially leading to some of the agent passing through the shunt entirely. The material could also prompt recanalization through blockage of the feeder artery as well as accumulating subcutaneously or intramuscularly, leading to infection [40]. Figure 5 shows the use of nBCA to embolize an upper extremity AVM.

Onyx and nBCA differ in their manner of deployment. Onyx can be injected into the site of the nidus without immediate precipitation, like nBCA, instead taking about 5 min to fully solidify [41]. Unlike nBCA, Onyx is not adhesive, meaning that the injection times for Onyx will be longer, with the ability to cease embolization if needed [39]. However, Onyx requires catheters compatible with dimethyl sulfoxide (DMSO), a toxic substance to humans [38,41]. The use of these two embolic agents is based on operator choice and experience. As the Onyx procedure is technically more intensive, it requires a more skilled interventionalist to successfully perform the procedure.

**Figure 6:**
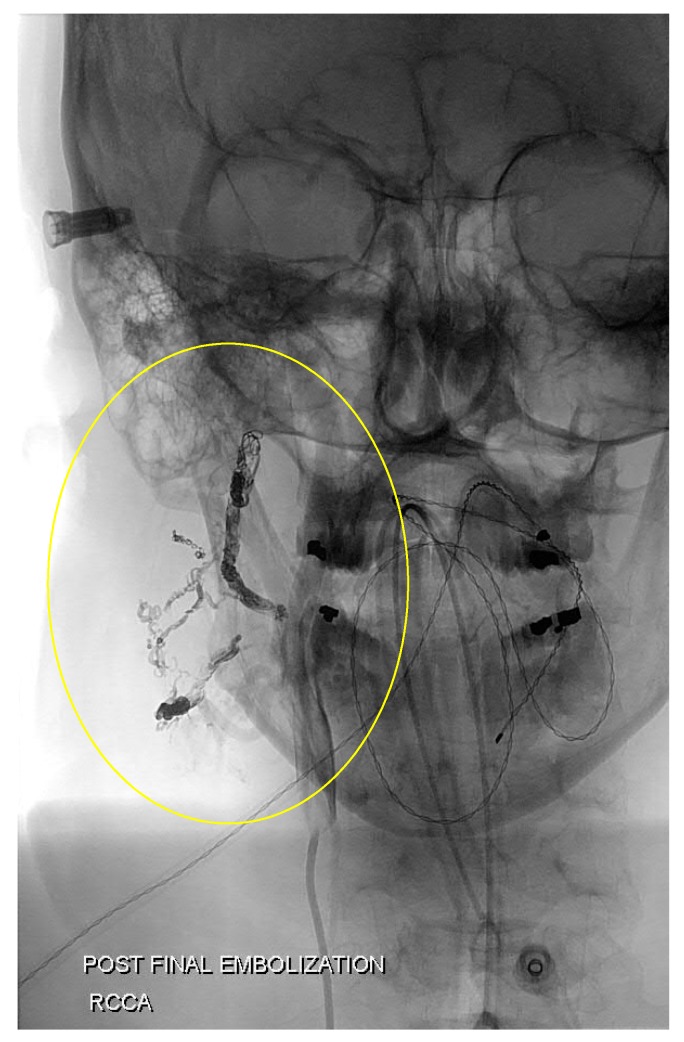
Coil embolization of a neck AVM. Coils were placed in the feeder arteries to this nidus that branched mainly from the right occipital artery.

Coiling is another embolic procedure that occludes vessels with compacted wire that induces thrombosis in the coiled vessel. Figure 6 shows the use of coils to embolize an AVM in the neck and face. Blood flow slows enough to cause clot formation. Coils inserted into vessels are recommended to be 20–30% greater than the vessel diameter to prevent the possibility of coil migration and distal embolization [38]. Pushable coils are typically used in the field of interventional radiology. These coils must physically be pushed out of the catheter into the target vessel, and this can lead the catheter to back out of its position in the vessel and cause nontarget embolization. Once a pushable coil is deployed, it cannot be repositioned [38]. Detachable coils have the benefit of better control as they are only detached after assessing the stability of the coil(s) upon deployment. If they are unstable as deployed, they can be retrieved rather than permanently detached.

Similar to coil embolization, Amplatzer plugs are dependent on the patient’s coagulative abilities and are therefore less effective in patients that have coagulopathy. These plugs are straight disks that are placed in the vessels to halt the flow of blood and initiate thrombosis. They can allow for precise placement, however, they need to be inserted into straight portions of vessels and may take several minutes to initiate thrombosis in high flow vasculature, such as AVMs [38].

### 5.3. Preoperative and Supportive Use of the Transarterial Approach

The arterial treatment of AVMs can also be used as an adjunctive approach, in which the goal is not to directly embolize the nidus, but instead optimize success for a subsequent approach to completely occlude the AVM. The transarterial occlusion of feeding vessels into the nidus can create temporary local hypotension. This in turn can allow the administration of embolic agents via transvenous or direct cutaneous approaches with a longer dwell time because of the reduced high-flow nature of the nidus. There have been multiple reported cases in which transarterial approaches to AVM provided the pre-procedural improvement of the hemodynamics of the nidus [24,25,26,27].

The transarterial treatment of AVMs has been reported as a technique to increase the chance of success of transvenous retrograde AVM embolization (TRAE) [24]. The aim of the transarterial use of the agents in this procedure is two-fold. First, coiling and embolization, either in the feeding arteries or within the nidus, reduce the flow of blood into the nidus. In this case, the purpose of arterial treatment is to create temporary hypotension to reduce the high-flow nature within the AVM. Secondly, transarterial occlusion before TRAE reduces the risk of the embolic agents entering normal venous circulation and causing distal embolization [24]. These embolic agents can then have maximal effect on the nidus because they do not rapidly pass through the collection of vessels or become diluted through high-flow circulation. This highlights the importance and versatility of the transarterial catheter approach as a means of reducing the nidus flow or size in order to maximize the effect of other treatment approaches.

A similar use of transarterial embolization preoperatively has been noted to embolize a ruptured utero-ovarian AVM with the use of Onyx [25,26]. Again, transarterial catheterization and embolization is the preferred method, but can be difficult to achieve if the arterial vessels into the nidus are too tortuous. The transvenous approach would be easier but increases the risk for non-target embolization and increased pressure within the nidus due to a retrograde approach. This increase in pressure could possibly cause nidus rupture. A combination of treatment approaches was used in this patient case. The transarterial approach was used to first reduce the blood flow through the draining vein so that the transvenous embolization of the nidus was safer and more effective [25,26].

Specifically in brain AVMs, balloon occlusion through transarterial access as well as transarterial nBCA or Onyx embolization have been used to create local hypotension and reduce the flow through the nidus [27]. The use of transarterial approaches to decrease the flow of blood through the nidus are particularly useful in larger AVMs, where there would be a higher number of arterial connections feeding into the nidus. Transarterial embolization would embolize the connections and reduce the intra-nidal flow sufficiently enough to allow TRAE to be successful [24]. The use of balloon occlusion to reduce the flow within the nidus has also been recommended for the treatment of renal AVMs, so that the agent of treatment can remain in the nidus instead of being washed downstream due to the high-flow nature of the AVM.

However, caution must be taken with these approaches, as it has been noted that coiling in feeding arteries could block access to the nidus and actually recruit additional vessels into the nidus, perhaps worsening the prognosis of the AVM [6]. Pre-procedure hypotension could also be achieved at the systemic level through the use of vasodilators during anesthesia, such as sodium nitroprusside, esmolol, or isoflurane [24].

## 6. Risk of Arterial Treatment of AVMs

There has been much emphasis placed on understanding and evaluating the angioarchitecture of the feeding and draining vessels of a nidus, because it can affect the success of an approach in embolizing the AVM. There are multiple risks involved with the transarterial treatment of AVMs that must be considered. These risks include ischemic damage to the surrounding tissue, recanalization of the nidus due to incomplete embolization, nontarget embolization downstream of the nidus, and possible rupture bleeding. Careful consideration of the risks against the possibility of treatment success is important to determine whether a patient’s condition can be cured or simply managed.

One risk of the complete embolization of the AVM is possible ischemic damage to the surrounding tissue. Imaging should be used to evaluate the relevant geography of the tissue around the nidus to determine the risk of ischemia following embolization. Complete embolization of the nidus can also potentially cause the rupture of the AVM due to sudden hemodynamic pressure changes [42].

Embolotherapy still has a risk of recanalization if the nidus is not completely occluded, prompting the recruitment of additional vessels. As an alternative, the ablation of AVMs is also performed using absolute ethanol. Recurrence and vessel recruitment have not been observed with ethanol ablation [3], because of the destruction of the nidus endothelium. Endothelial injury prompts a strong response in the clotting cascade, thrombosing the area. However, this dangerous sclerosing agent can cause significant damage if it enters normal circulation. Therefore, pulmonary artery pressure must be strictly monitored to ensure no entrance of ethanol into pulmonary circulation [3]. The sclerosing agent could prompt the development of a pulmonary embolism, inducing hypoxemia and decreased respiratory function in the patient due to the embolus preventing deoxygenated blood from reaching capillaries in the lung [43].

It is possible for AVMs to recanalize after a method of treatment due to angiogenic behavior that recruits additional feeding vessels to the nidus and preserves the abnormal connection between the arterial and venous circulation. This can be caused by incomplete nidus occlusion and embolization of only the main feeder arteries, but not all feeder arteries. Though flow may be reduced temporarily, if there is no additional attempt at embolization via transvenous or direct cutaneous approaches, the patient’s clinical presentation can worsen because angiogenesis can exacerbate the AVM. There have been reports of the recurrence of AVMs after coil embolizations in mandibular AVMs [44], perhaps due to partial embolization causing recanalization. Angiogenesis has known to be promoted by endothelial cells that release vascular endothelial growth factor (VEGF) under hypoxic conditions to recruit endothelium for new vessels, as a means to receive more oxygen with increased vasculature [45]. Perhaps partial embolization of the nidus prompts endothelial cells in the AVM to react to a decrease in oxygen supply, thereby causing the recurrence and possible worsening of the patient’s clinical presentation.

Due to the pressure gradient difference caused by the nidus, it is possible for embolic agents to pass rather quickly through the low-pressure cell. This can lead to the unintended embolization of vessels downstream from the nidus [21]. Unintended downstream embolization can cause thrombosis in normal vasculature, leading to conditions such as myocardial infarction, limb ischemia, pulmonary embolism, and stroke, depending on the region of the nontarget embolization [46,47]. Flow-reduction techniques can decrease the probability of these agents passing into normal venous circulation.

This risk is present even with pushable coil embolizations, as the high flow nature of the AVM can perhaps dislodge coils meant to embolize and send them downstream. The risk of coil embolization failure increases in patients with coagulopathies or patients taking anticoagulation medications. The lack of occlusion can perhaps lead to the rupture of the vessels near the nidus [36].

Ethanol sclerotherapy via the transarterial approach into the nidus has its own risks as well. There is a risk of the shunting of the agent to the lungs, causing pulmonary hypertension, as mentioned previously, as well as other potential complications for the patient. Localized skin necrosis and tissue damage have also been observed using this approach, as ethanol is a highly reactive and damaging agent [48]. Rapid dilution, especially in high-flow malformations, can also cause unintended infarction of the tissue downstream [39]. The downsides of this treatment, as well as other approaches, can sometimes be avoided by combining multiple approaches, embolizing to reduce flow such that the sclerosing effect of the ethanol remains as much with the nidus as possible [19].

## 7. Treatment Effectiveness

Nidus morphology combined with the choice of treatment affects the success of AVM occlusion. A treatment can be considered effective if it is able to minimize the chance of recanalization, recurrence, or progression of the nidus. A completely successful treatment of AVMs involves the complete embolization of the nidus along with occlusion of all feeder arteries [33]. Otherwise, this can prompt the reappearance of the nidus with new feeder vessels over time [33].

Nidus morphology can determine the effectiveness of certain treatment approaches because the angioarchitecture affects flow dynamics and how the embolic agent interacts. For example, liquid embolics are not as effective in AVMs where the morphology of the nidus is predominantly a large outflowing draining vessel, because the high flow nature into the venous side increases the risk of distal embolization. The Yakes classification proposes the coiling of the draining vessels to decrease the high flow nature before attempting to treat the nidus for the optimal effect [8]. In morphologies that are more infiltrative, such as Yakes type IV AVMs, liquid embolic treatment cannot enter smaller vessels, therefore making ethanol sclerotherapy more effective. There is a risk of tissue necrosis, however, with this treatment [8].

Treatment with the liquid agent Onyx was developed as an effective tool specifically against cerebral and dural AVMs, where ethanol sclerotherapy posed too great a risk of seizures and tissue necrosis in functional regions. As the previous literature reports, the agent was adapted for use in AVMs of the body. The agent showed promise for complete occlusion in short-term angiography. However, there has been recent literature stating the ineffectiveness of Onyx in preventing recanalization of the nidus. Hoss et al. conducted a retrospective review of 18 onyx embolizations of high-flow craniofacial AVMs and identified histopathologic signs of vascular recanalization proximal to the Onyx cast material in 13 of the specimens [37].

The transarterial approach to AVM treatment has shown its effectiveness as a supportive treatment to other procedures targeting the nidus. A study by Lv et al. demonstrated that the TRAE technique, when supplemented with transarterial embolization beforehand, completely obliterated AVMs in 56 out of 60 patients (93.3%) [24]. This indicates that in some cases the transarterial approach may be more effective simply in a supportive role, to merely reduce the size of the nidus. In these cases, due to morphology, transarterial embolization alone would not work because the other feeder arteries would contribute compensatory increased perfusion to the nidus over the time of the follow up.

The previous literature supports the use of ethanol sclerotherapy as a long-term effective treatment with minimal signs of recanalization, angiogenic recruitment, or progression. Ethanol as a sclerotic agent ablates the nidus by causing inflammation of the nidus endothelium and prompting intense thrombosis [44]. This injury prevents the release of angiogenic factors that prompt the recruitment of new feeder vessels to the nidus site [3]. Instead of simply blocking flow into the nidus, the ethanol agent destroys the functional endothelium, increasing the probability of curing AVMs [49]. In a study by Cho et al., 66 patients with inoperable AVMs were retrospectively analyzed to determine the efficacy of ethanol embolization on different morphologies in the Cho–Do classification system. Ethanol embolization was most effective for AVMS of type II (100%) followed by type IIIb (83%) [7]. In a study by Do et al., symptoms from and clinical outcomes of AVMs were completely resolved in 23 out of 40 (58%) of patients who underwent ethanol embolization [40].

## 8. Conclusions

Arterial embolization of the nidus can play many versatile roles in treating arteriovenous malformations of the body. High importance is given to classifying the AVM based on its morphological structure before applying any treatment path to accurately assess the probability of success for each therapeutic approach. The orientation of feeder vessels as well as other individual characteristics of the nidus will affect the difficulty of arterial treatment, and perhaps make it necessary to use another treatment approach. Even if the imaging workup determines that arterial treatment would not completely embolize the nidus, it is valuable as a preoperative tool to reduce an AVM’s nidal flow and diminish its high-flow nature. This can possibly increase the chance of embolization success for transvenous or direct percutaneous approaches in successful embolization.

## Figures and Tables

**Table 1:** Cho–Do classification system of arteriovenous malformations based on nidus morphology [7].

Nidus Type	AVM Label [6]	Morphological Structure [6]
I	arteriovenous	Three arteries or less shunting to a single vein
II	arteriolovenous	Multiple arterioles shunting to a single vein
IIIa	non-dilated arteriolovenulous	“Blush” of connections between arterioles and venules
IIIb	dilated arteriolovenulous	Complex vascular network between arterioles and venules

**Table 2:** Yakes AVM classification system [8].

Nidus Type	Description	Treatment Approach
Ia	Direct AVF	Mechanical occluding device
IIa	Typical AVM nidus	Transcatheter and direct puncture ethanol embolization
IIb	AVM nidus shunting into aneurysmal vein	Same as type IIa as well as coiling outflow vein
IIIa	Aneurysmal small vein where nidus resides in vein wall with single outflow vein	Coiling single aneurysmal outflow vein
IIIb	Type IIIa with multiple outflow veins	Coiling each outflow vein
IV	Tissue infiltrative AVM	Transcatheter or direct puncture embolization

**Table 3:** Schobinger staging system [10].

Stage Number	Clinical Presentation [2]
I	Dermatologic Symptoms—cutaneous blush
II	Bruits and thrills
III	Pain, ischemic necrosis, ulceration
IV	High-output cardiac failure

**Table 4:** Absolute and relative indications of treatment for AVMs [19].

Absolute Indications	
	Hemorrhage
	High-output cardiac failure
	Chronic venous hypertension
	Lesions at limb or life-threatening location
Relative Indications	
	Disabling pain
	Functional disability
	Cosmetic deformities
